# Proteometabolomic response of *Deinococcus radiodurans* exposed to UVC and vacuum conditions: Initial studies prior to the Tanpopo space mission

**DOI:** 10.1371/journal.pone.0189381

**Published:** 2017-12-15

**Authors:** Emanuel Ott, Yuko Kawaguchi, Denise Kölbl, Palak Chaturvedi, Kazumichi Nakagawa, Akihiko Yamagishi, Wolfram Weckwerth, Tetyana Milojevic

**Affiliations:** 1 Department of Biophysical Chemistry, University of Vienna, Vienna, Austria; 2 School of Life Sciences, Tokyo University of Pharmacy and Life Sciences, Tokyo, Japan; 3 Department of Ecogenomics and Systems Biology, University of Vienna, Vienna, Austria; 4 Graduate School of Human Development and Environment, Kobe University, Kobe, Japan; 5 Vienna Metabolomics Center (VIME), University of Vienna, Vienna, Austria; University of Alabama at Birmingham, UNITED STATES

## Abstract

The multiple extremes resistant bacterium *Deinococcus radiodurans* is able to withstand harsh conditions of simulated outer space environment. The Tanpopo orbital mission performs a long-term space exposure of *D*. *radiodurans* aiming to investigate the possibility of interplanetary transfer of life. The revealing of molecular machinery responsible for survivability of *D*. *radiodurans* in the outer space environment can improve our understanding of underlying stress response mechanisms. In this paper, we have evaluated the molecular response of *D*. *radiodurans* after the exposure to space-related conditions of UVC irradiation and vacuum. Notably, scanning electron microscopy investigations showed that neither morphology nor cellular integrity of irradiated cells was affected, while integrated proteomic and metabolomic analysis revealed numerous molecular alterations in metabolic and stress response pathways. Several molecular key mechanisms of *D*. *radiodurans*, including the tricarboxylic acid cycle, the DNA damage response systems, ROS scavenging systems and transcriptional regulators responded in order to cope with the stressful situation caused by UVC irradiation under vacuum conditions. These results reveal the effectiveness of the integrative proteometabolomic approach as a tool in molecular analysis of microbial stress response caused by space-related factors.

## Introduction

The Gram positive bacterium *Deinococcus radiodurans* is extremely resistant to several environmental conditions, such as ionizing radiation [1], UV radiation [2], oxidation stress [3] and desiccation [4]. Such a multifaceted resistance of *D*. *radiodurans* ensures its potential to survive in the harsh outer space environment during interplanetary transfer. The Tanpopo, which means dandelion in Japanese, mission [5] includes a long-term exposure (separate experiments between one to three years) of *D*. *radiodurans* on the Japan Experimental Module of the International Space Station (ISS) in the low Earth orbit (LEO). It is performed in order to validate the panspermia theory [6]—the possible transfer of life between Earth and extra-terrestrial bodies. To ensure that *D*. *radiodurans* is suitable for a long term exposure experiment on the ISS, several preliminary exposure experiments have been performed by Kawaguchi, Yang [7]. During these experiments, the different parameters (heavy ion beam radiation, temperature cycles, vacuum and UVC radiation) were adapted to mirror LEO conditions and the following survival tests revealed that UVC radiation had the highest impact on cell survivability [7, 8]. It was shown that, even though *D*. *radiodurans* possesses high tolerance against UVC radiation, direct exposure of monolayers to LEO conditions results in no survival [5]. However, aggregated *Deinococci* cells exposed to UVC radiation showed that they should withstand solar UV radiation on the ISS for one year as multilayers of dehydrated cells, and survive, wherein upper cellular layers cover and protect underlying inner cells. Approximately 200 μm of cell layers are necessary to shield the inner layers of *D*. *radiodurans* efficiently from solar UV radiation. Based on these findings, massapanspermia has been proposed, implying that apart from rocks which shield the microbes against solar UV radiation (i.e., lithopanspermia), it is possible for cell-aggregates to function as a protective ark for interplanetary transfer of microbes, where upper layers shield lower layers from the harmful environment [7, 8]. Proving this theory is a part of the Tanpopo mission, as cell aggregates with different thicknesses of *D*. *radiodurans* are directly exposed to LEO conditions. These factors are microgravity, vacuum down to 10^−7^ Pa, solar UV radiation, galactic radiation, solar cosmic radiation, van Allen Belts and temperature cycles (from -120°C up to 120°C every 90 min) [9].

Complementing survivability studies, an approach to unravel the response to LEO conditions on a molecular level is desirable, as it might provide an explanation how it is possible for certain organisms to survive under such extreme conditions. A systems biology approach, especially the combination of several–omics analysis, improves the knowledge of microbial stress response mechanisms and explains how microorganisms respond to environmental changes on the molecular level. Environmental stresses can damage cells due to the formation of reactive oxygen species (ROS), which cause lipid peroxidation, protein oxidation and oxidative DNA damage. Exogenous factors can further interfere with genome integrity as they cause double strand breaks, primarily induced by vacuum and single strand breaks, primarily induced by UVC irradiation [3]. In addition to breaks, three major classes of bipyrimidine photoproducts (BPPs), cyclobutane pyrimidine dimers, pyrimidine 6–4 pyrimidone photoproducts and Dewar isomers, are formed if organic material is exposed to UVC radiation [10]. Although there is no evidence that the DNA damage repair mechanism is very different in *D*. *radiodurans* compared to *Escherichia coli* [11]. Despite the number of BPPs after UVC irradiation of 500 Jm^-2^ being comparable between *D*. *radiodurans* and *E*. *coli*, *D*. *radiodurians* is about 25 times more resistant to BPPs compared to *E*. *coli* [12, 13]. The reason for this higher resistance lies in the protection of intracellular proteins against UV induced oxidative damage [3]. However, as the amount of DNA damage caused by UVC irradiation and desiccation is severe, an efficient DNA repair mechanism is still important. Two separate nucleotide excision repair pathways act simultaneously to remove BPPs [14]. The pathways rely on the proteins UV DNA damage endonuclease (*uvsE*) and UvrABC system protein A (*uvrA*) [13]. Both pathways require the proofreading DNA polymerase I (*polA*), as mutants without the *polA* gene are extremely sensitive to UVC irradiation [15]. Another essential protein for enzymatic repair of DNA damage is RecA, which cleaves the repressor LexA that represses SOS response genes, like DNA repair enzymes [16]. After successful excision, recombinational repair is performed. The genome repair does not rely on a new pathway for double-strand break repair, caused by desiccation stress, but is rather a set of recombinational DNA repair functions which can be observed in many other species [17]. Important proteins for the recombination process are gyrases (*gyrA* and *gyrB*), which cause negative supercoils to favor strand separation, DNA replication, transcription, recombination and repair [18]; PprA to stimulate the end-joining reaction catalyzed by DNA ligases [19] and the different single-stranded DNA binding proteins DdrA [20], DdrB [21], DdrC [22] and DdrD [22] for RecA independent genome reconstruction processes.

The aim of this study was to decipher the molecular response of *D*. *radiodurans* to space-related conditions of UVC radiation and vacuum using the experimental set-up of Tanpopo orbital project. Here we present an integrative proteometabolomic approach applied to reveal key components of the molecular mechanism of *D*. *radiodurans* survivability in response to UVC irradiation under vacuum conditions.

## Materials and methods

### Cultivation and preparation of dehydrated *D*. *radiodurans* cells

*D*. *radiodurans* R1 (ATCC 13939) was cultured 15 h in mTGE medium (1%(w/V) tryptone, 0.6%(w/V) beef extract, 0.2%(w/V) glucose) at 30°C in an incubator with shaking speed of 150 rpm until it reached the anaphase of the logarithmic phase. Liquid cultures of *D*. *radiodrans* R1 were washed in 10 mM phosphate buffer (PB). This step was repeated three times. Aluminum plates containing cylindrical wells (2.0 mm diameter, 2 mm depth) with flat floor were used as sample holders [8]. Twelve microliter of a cell suspension (2.9*10^9^ cells/mL) were dropped into 4 wells and dried up under 3.3*10^−2^ atm in a desiccator at room temperature under sterile conditions. These steps were repeated 6 times. The amount of deinococcal cells was 3.5*10^7^ cells per well corresponding to a multilayer of 200 μm thickness (S1 Fig). The cells were dried up under 3.3*10^−2^ atm for 16 h.

### UVC and vacuum exposure

A mercury lamp 254 nm was used to irradiate deinococcal cells in the vacuum chamber. The setup of the UVC-irradiation experiment was described previously [7]. The aluminum plates containing dehydrated cells of *D*. *radiodurans* were exposed to UVC_254 nm_ dose 862.0 kJ/m^2^ under approximately 400 Pa. Control samples were only dehydrated cells kept in a desiccator at room temperature.

### Survival assay

After the exposure to UVC and vacuum, cells were recovered from wells of aluminum plate using PB 10 mM. The cell suspension was serial diluted with PB 10 mM and the diluted cell suspension was dropped on mTGE agar plates [7]. The plates were incubated at 30°C for 1.5 days. Surviving fractions were determined from the ratio of *N*/*N*_0_, with *N* being the number of colony formation unit (cfu) of the irradiated cells and *N*_0_ being the CFU of the control samples.

### Scanning electron microscopy

The morphology and cellular integrity of the dehydrated cells of *D*. *radiodurans* deposited on aluminum plates were examined with a Zeiss Supra 55 VP scanning electron microscope. The dehydrated cells were coated with a thin Au/Pd layer (Laurell WS-650-23 spin coater). The imaging of dehydrated clustered cell layers and single cells was performed with the acceleration voltage of 5 kV.

### Cultivation conditions

For cultivation of the dehydrated *D*. *radiodurans* cells, two wells were resuspended in 100 μL phosphate buffer (10 mM K_2_HPO_4_, 10 mM KH_2_PO_4_, pH 7) to inoculate 10 mL of mTGB medium. In total 4 biological replicates of the control non-irradiated cells and 4 biological replicates of the UVC/vacuum-irradiated cells were incubated at 30°C with an agitation rate of 150 rpm for 5 hours. The growth of the cells was monitored by cell counting using a hemacytometer.

### Integrative extraction of proteins and metabolites

Extraction and analysis of metabolites and proteins from one sample was performed according to Weckwerth, Wenzel [23] with slight modifications (for a detailed version of the extraction protocol see dx.doi.org/10.17504/protocols.io.j3bcqin). The cells were harvested (3000 g, 5 min, 4°C), washed with 10 mM phosphate buffer three times and finally resuspended in ice-cold 1 mL MCW (methanol:chloroform:water 2.5:1:0.5). 0.5 g of FastPrepTM lysing matrix B (MP Biomedicals, Santa Ana, USA) was added to the mixture and the cells were homogenized with a FastPrepTM-24 Instrument (MP Biomedicals, Santa Ana, USA) at 3x4.5 m/s for 30 s with a 5 min cooldown on ice between the cycles. After centrifugation (21000 g, 15 min, 4°C) the supernatant, which contained the metabolites was transferred into a new tube. The pellet, which contained the precipitated proteins was stored at 4°C for the subsequent extraction. Phase separation was induced by adding 200 μL of water. The phases were separated in different tubes and dried in a vacuum concentrator.

### Derivatisation and analysis of the metabolites with GC-TOF-MS

Polar metabolites were dissolved in 10 μL of 40 mg mL-1 methoxyamine-hydrochloride in pyridine through shaking at 650 rpm at 30°C for 90 min. Subsequently, 40 μL of a silylation mix (1 mL N-methyl-N-trimethylsilyltrifluoroacetamid spiked with 30 μL of a mix of even-number alkanes (C10-C40)) was added and the mixture was incubated for 30 min at an agitation rate of 650 rpm at 37°C. After centrifugation (14000 g, 2 min), the supernatant was transferred into a glass vial and 1 μL of it was injected into the GC (Agilent® 6890 gas chromatograph) in splitless injection mode.

For separation of the metabolites, an Agilent HP-5MS column (30 m length, 0.25 mm diameter and 0.25 μm film) was used. Further parameters were set as following: flow rate 1 mL min^-1^; injection temperature 230°C; column temperature started at 70°C for one minute, then heated up to 330°C in 9 min, where it was hold for 8 min; recorded masses in the LECO Pegasus^®^ 4D GC×GC-TOF spectrometer were set between 40–700 m/z. Apart from the samples, a house intern standard mix of certain metabolites was measured to get level 1 identifications of common primary metabolites.

Identifications of the metabolites were based on matching the obtained MS-spectra and retention times with an in-house library (extended gmd database). Peak integration was performed with the LECO ChromaTOF^®^ software. Metabolites which were also identified in the standard mix were considered a level 1 identification, the ones which were not present in the mix, but the retention index and the mass spectrum was similar to one of the database were considered a level 2 identification. The measured areas were normalized against the number of cells, used for the extraction.

### Protein extraction

The pellets were suspended in 400 μL of a protein extraction buffer (100 mM NaCl, 100 mM Tris-HCl pH 7.5, 10% (v/v) Glycerol, 3% SDS (m/v)) and an equal amount of phenol (saturated with Tris-HCl, pH 7.0, Roth) was added to the suspension. The mixture was vortexed, centrifuged (20000 g, 2 min, 4°C) and the lower, phenolic phase was transferred into a new tube. To precipitate the proteins, five volumes of ice-cold 0.1 M ammonium acetate in methanol was added. After keeping the suspension at -20°C overnight, it was centrifuged (5000 g, 30°C, 4°C) and the pellet was washed twice with methanol and once with acetone.

### Protein quantification and In-gel digestion

Protein analysis was performed according to Chaturvedi, Ischebeck [24] with slight modifications. The pellet was dissolved in approximately 30 μL of urea buffer (6 M urea, 5% SDS). The proteins were quantified with a BCA (bicinchoninic acid) assay kit with a BSA standard. A total amount of 100 μg protein for each sample was mixed with 5x Laemmli buffer, heated at 95°C for 5 min and applied on a SDS-polyacrylamide gel (separation gel 12%, stacking gel 5%). A voltage of 40 V was applied until the samples reached the interphase between the gels. Then the voltage was switched to 80 V until the bromophenol blue run approximately one centimeter into the separation gel. Gel staining was performed with 40% (V/V) methanol, 10% acetic acid (V/V), 0.1% (w/V) Coomassie R-250 in milliQ-water for 30 min, followed by four 20-min destaining (40% (V/V) methanol, 2% (V/V) acetic acid). Finally, the gel was washed in milliQ-water for half an hour and all protein lanes for each replicate were cut out of the gel.

For further analysis, the gel bands were cut into small pieces around 1 mm^3^ and 1 mL 200 mM AmBic (ammonium bicarbonate) in 50% ACN (acetonitrile) solution was added to each replicate. The samples were incubated (37°C, 30 min, agitation rate 650 rpm) and the supernatant was discarded. This process was repeated until the colour of the gel pieces completely disappeared. Afterwards, 500 μL of 50 mM AmBic in 5% ACN were added, incubated (37°C, 15 min, agitation rate 650 rpm) and the supernatant was discarded. Finally, 500 μL of ACN were added to the gel pieces, incubated (37°C, 10 min, agitation rate 650 rpm) and the supernatant was discarded. Gel pieces were air-dried and 12.5 ng/μL trypsin (Roche; in 25 mM AmBic, 10% ACN, 5 mM CaCl2) was added until all gel pieces were covered by the solution. Tryptic digestion took place at 37°C for 16 h without shaking.

### Peptide extractions and desalting

For the peptide extraction, 150 μL of 50% ACN with 1% formic acid were added to each tube, incubated for 5 min at room temperature, sonicated shortly in a low intensity ultrasound bath and the supernatant was transferred to a new tube. The procedure was repeated once. Ultimately, 100 μL 90% ACN with 1% formic acid were added, incubated 5 min at room temperature and the supernatant was transferred to the same tube again. Extracted peptides were dried down in a vacuum concentrator.

The peptides were suspended in 4% ACN, 0.25% formic acid and applied on C18-Bond Elut 96-well plates (Agilent Technologies). They were washed five times with 400 μL of water, whereby the first flow through was kept for another desalting step with graphite. Washed peptides were eluted with 400 μL methanol. Graphite spin column (MobiSpin Column F, MoBiTec) desalting with the first flow through was performed according to the manufacturer’s manual (Thermo scientific, Pierce® graphite spin columns). The desalted eluates from the plates and the columns were combined for each sample and dried down in a vacuum concentrator.

### Shotgun proteomics with HPLC nESI-MS/MS

Peptides were dissolved in 2% ACN with 0.1% formic acid to a theoretical concentration of 0.2 μg μL^-1^ based on the amount of protein which was loaded on the gel. 1 μg of each sample (4 biological replicates for UV and control) was applied on a C18 reverse phase column (Thermo scientific, EASY-Spray 500 mm, 2 μm particle size). Separation was achieved with a 180 min gradient from 100% solution A (0.1% formic acid) to 40% solution B (90% ACN and 0.1% formic acid) with a flow rate of 300 nL min^-1^. nESI-MS/MS measurements were performed on an Orbitrap Elite (Thermo Fisher Scientific, Bremen, Germany) with the following settings: Full scan range 350–1800 m/z resolution 120000, max. 10 MS2 scans (activation type CID), repeat count 1, repeat duration 30 sec, exclusion list size 500, exclusion duration 30 sec, charge state screening enabled with rejection of unassigned and +1 charge states, minimum signal threshold 500.

### Protein identification and LFQ (label free quantification)

For identification, a Uniprot database (last updated 2015-06-20) containing the annotation of 3088 proteins for *D*. *radiodurans* was used. The received Thermo raw files from the instrument were identified and quantified in MaxQuant (version 1.5.7.0) with the following parameters: first search peptide tolerance 20 ppm; main search peptide tolerance 4.5 ppm; ITMS MS/MS match tolerance 0.6 Da; a minimum of 7 amino acid were required for the peptide identification and a minimum of two peptides for the protein identification; a maximum of two missed cleavages were allowed; a maximum of five modifications (oxidation of methionine and acetylation of the N-term) were allowed per peptide; a retention time window of 20 min was used to search for the best alignment function and identifications were matched between runs in a window of 0.7 min; a revert decoy database was used to set a cut-off at a FDR of 0.01. LFQ with a minimum ratio of two was performed when at least one MS2 identification was present.

### Statistical evaluation

Key metabolite pathways and protein abundance differences between the control cells and the cells exposed to UVC/vacuum conditions was analyzed with Perseus. PCAs and heatmaps were created with the R packages heatmaps.2 and ggplots. Cytoscape was used for the combined analysis of metabolomics and proteomics data. For all analysis, the LFQ intensity values which were calculated by MaxQuant, were used. First, the fold changes between the proteins were calculated. Proteins which weren’t identified in at least three of the four replicates in at least one condition were excluded from the list. After z-transformation of the values, a Welch’s T-test was performed between the two conditions. For all proteins with known annotations, different gene ontologies (cellular compartment, biological process, molecular function) and KEGG pathways were added as categorical columns. With these columns, a Fisher exact test (p-value < 0.02) was performed to identify gene ontologies/KEGG pathways with an unusual representation of proteins within the T-test.

## Results

### Effects of UVC/vacuum conditions on cellular integrity, growth and survivability of dehydrated *D*. *radiodurans*.

**Fig 1:**
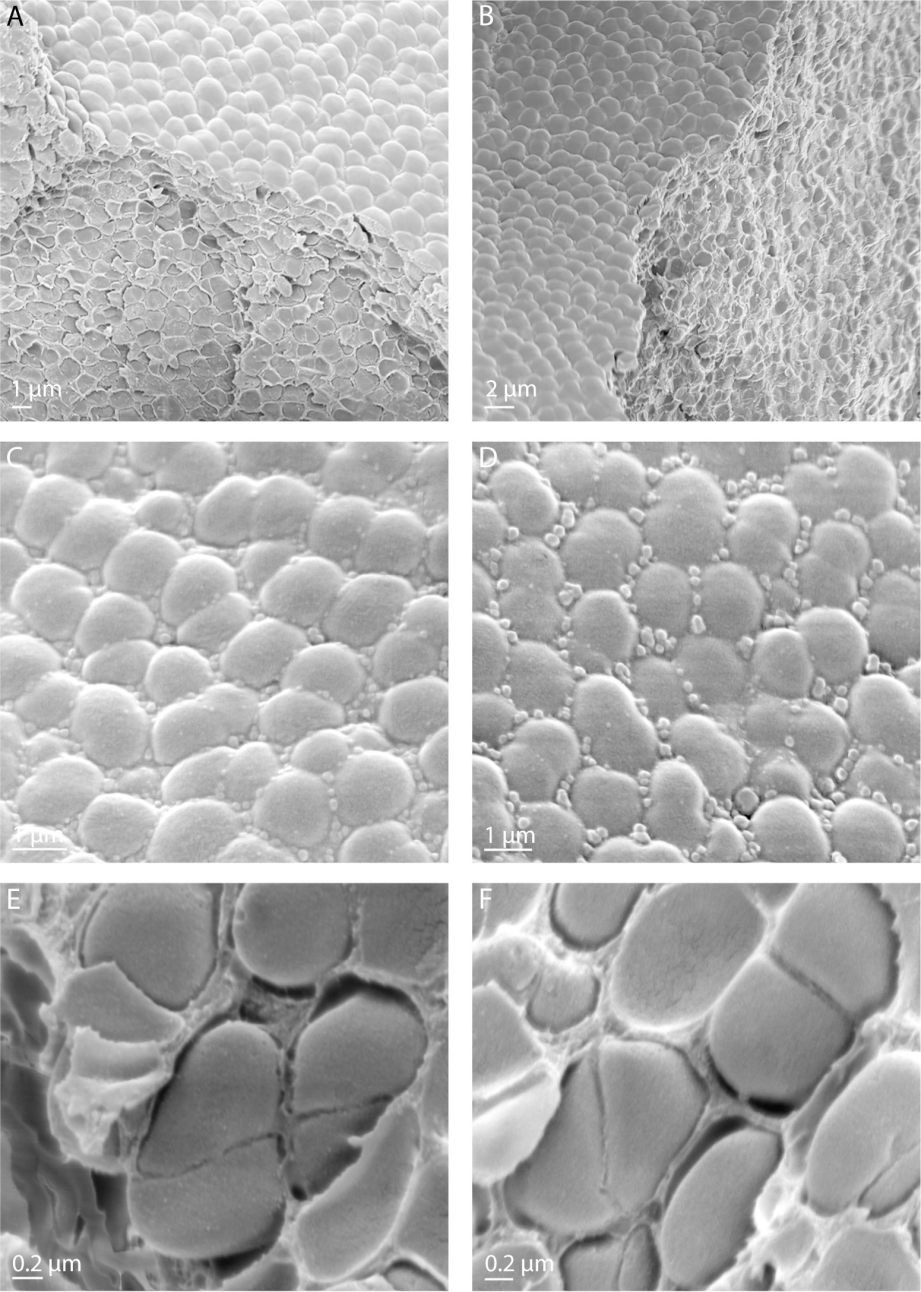
Scanning electron microscopy images of dehydrated cells of *D*. *radiodurans* deposited on aluminum plates and used in experimental set up of Tanpopo mission. (**A**, **B**) Scanning electron microscopy images, showing upper surface and inner content of multilayers of dehydrated *D*. *radiodurans* cells deposited on aluminum plates. (**C**, **D**) Higher magnification images displaying upper surface of multilayers of dehydrated cells of *D*. *radiodurans*. (**E**, **F**) Magnified images of tetracocci and diplococci of *D*. *radiodurans* taken from the inner part of dehydrated multilayers. (**A**, **C**, **E**) control cells of *D*. *radiodurans*; (**B**, **D**, **F**) cells of *D*. *radiodurans* exposed to UVC-vacuum conditions.

Survival assays after exposure to UVC irradiation under vacuum showed an average survival rate of 6.5*10^−1^ (0.04 s.d.) compared to non-exposed control cells. In order to investigate cellular integrity after UVC irradiation under vacuum conditions, the surface of dehydrated clustered cell layers of *D*. *radiodurans* deposited on aluminum plates was examined with scanning electron microscopy (Fig 1 and S1 Fig). The observed typical morphology of diplococci and tetracocci of *D*. *radiodurans* is shown in Fig 1. In line with the extreme desiccation resistance of *D*. *radiodurans*, there was no detectable damage of cell surface and morphology of *D*. *radiodurans* observed after drying procedure under the control conditions (Fig 1A, 1C and 1E and S1 Fig). UVC irradiation under the vacuum conditions neither affected morphology, nor cellular integrity of dehydrated cells of *D*. *radiodurans* (Fig 1B, 1D and 1F and S1 Fig). Correspondingly, the analysis of survivability of cells using standard microbiological plating techniques and counts of colony forming units showed a relative survival rate of 65% for UVC/vacuum exposed cells compared to control conditions (S1 Table).

### Functional analysis of identified proteins of *D*. *radiodurans*

The LC-Orbitrap Elite^™^ measurements identified 1661 proteins in at least one sample, comprising 54% of *D*. *radiodurans* genome. 59 proteins were only found in at least one of the UV irradiated replicates.

**Fig 2:**
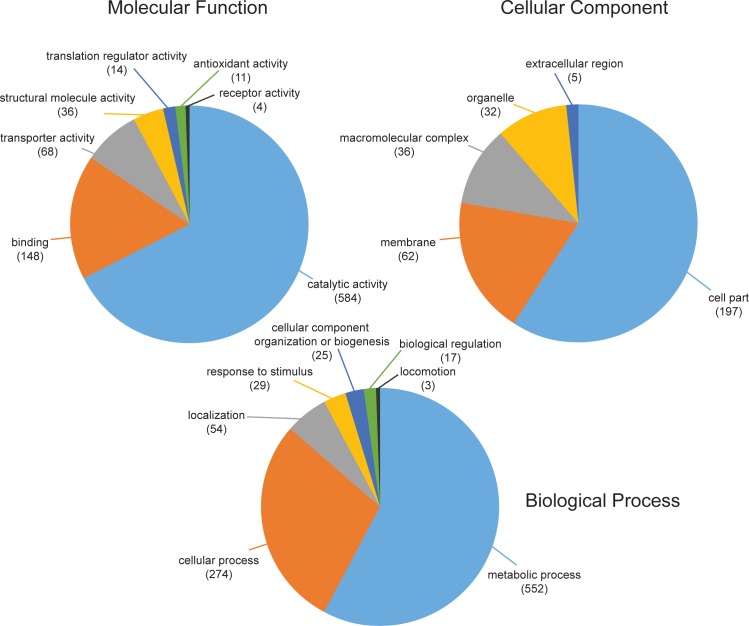
First two levels of gene ontology annotations of all proteins of *D*. *radiodurans*, which were identified in at least three out of four replicates in at least one condition.

GO (Gene Ontology) annotations were assigned using the PANTHER (Protein ANalysis THrough Evolutionary Relationships, http://pantherdb.org, V 11.1) online tool with the latest GO database (released 2017-04-24). The tool was able to map 1452 Uniprot IDs and provide the corresponding GO annotation in case there was one. In total the molecular functions of 865, the biological processes of 954 and the cellular compartments of 332 were annotated on the second hierarchical level of gene ontology annotations (Fig 2). Regarding their biological process, the three most dominant categories were metabolic process (38%), cellular process (19%) and localization (4%). 47% of the proteins from the category metabolic process belonged to primary metabolic processes. Overall, the most dominant protein classes were transferases (21%), hydrolases (16%), oxidoreductases (15%) and nucleic acid binding (12%). Apart from that, 553 proteins could be assigned to at least one KEGG pathway.

### Differences in the proteome between UVC/vacuum exposed and control cells

**Fig 3:**
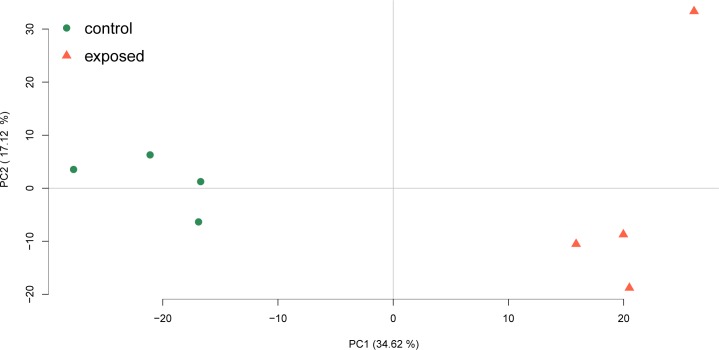
PCA score-plot of the z-scored label free quantification intensities. A clear separation can be observed on the PC1 level, which explains 34.62% of the data’s variance, between the UVC/vacuum treated samples (red) and the control samples (green).

For quantitative analysis, only proteins which were identified in at least three out of four replicates in at least one of the conditions were used (1457 in total) (S2 Table). The LFQ intensities were z-scored and the PCA-scores (Fig 3) for all four biological replicates showed a clear separation between control and UVC treated cells on component 1.

**Fig 4:**
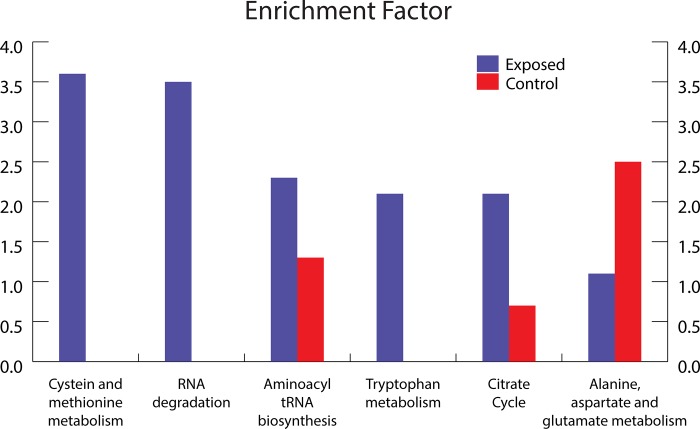
Bar plot of KEGG categories (x-axis) with corresponding enrichment factors (y-axis). **Categories with a minimum enrichment factor of two for either UVC/vacuum (blue) treated or control (red) conditions are mapped.** An enrichment factor of zero means that not a single protein in this category was upregulated in the displayed condition.

A Welch’s t-test (p-value < 0.05) identified 209 proteins as more abundant in the control cells and 357 in the cells exposed to UVC/vacuum conditions. With these proteins, a Fisher exact test for the KEGG categories was performed. The categories with an unusually high amount of proteins in one of the conditions are shown in Fig 4. Only categories with at least five identified proteins and a minimum enrichment factor of two in at least one condition are shown.

**Fig 5:**
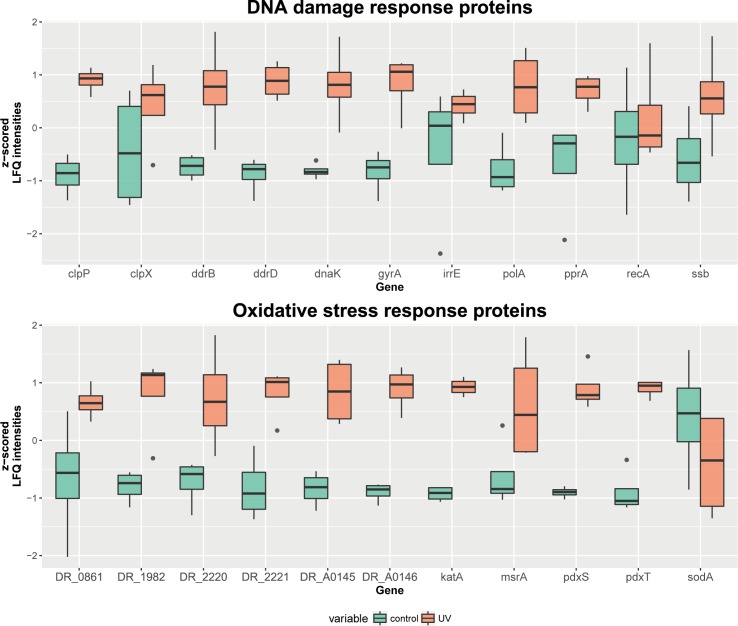
Boxplot of genes encoding important damage response proteins in *D*. *radiodurans* under the conditions of UVC/vacuum exposure. For every gene, the z-scored LFQ intensities are compared between the control and UVC/vacuum condition. The lower and the upper hinges correspond to the first and the third quartiles. The whiskers extend a maximum of 1.5 times the inter-quartile range. Outliers are indicated as dots. Proteins which are encoded by the mapped genes: Clp protease subunits (clpP and clpX), DNA damage response proteins (ddrB and ddrD), chaperone (dnaK), DNA gyrase subunit A (gyrA), radiation response metalloprotease (irrE), DNA polymerase (polA), DNA repair protein (pprA), recombinase (recA), single-stranded DNA-binding protein (ssb); catalase (katA), uncharacterized protein (DR_A0146), superoxide dismutase (sodA), phytoene dehydrogenase (DR_0861), Pyridoxal 5’-phosphate synthase (pdxS and pdxT), thioredoxin reductase (DR_1982), putative peroxidase (DR_A0145), peptide methionine sulfoxide reductase (msrA), tellurium resistance protein (DR_2220 and DR_2221).

Annotations and overrepresentation studies provide an overview of pathways which might be affected by the applied stress condition. However, the majority of proteins (> 99.2%; May 2017) in the gene ontology database are annotated based on automatic algorithmic sequence similarity search instead of manual curation. Therefore, a deeper comparison to the literature and described proteins is inevitable. Fig 5 shows boxplots of mainly manually curated proteins/genes related to DNA damage and oxidative stress response. Most of these proteins (8 out of 11 of selected DNA damage response proteins and 10 out of 11 of selected oxidative stress response proteins) show a significantly higher abundance in the UVC/vacuum exposed cells of *D*. *radiodurans*. Variances and number of outliers between the two conditions are similar for the chosen proteins.

### Metabolomic analysis of *D*. *radiodurans*

**Fig 6:**
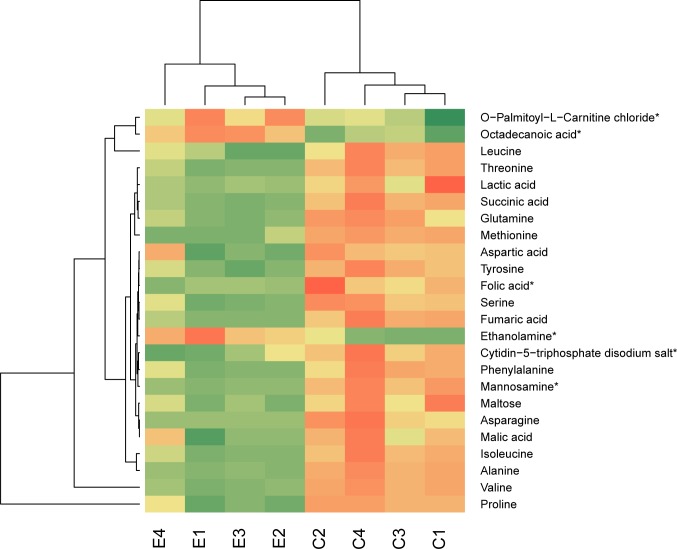
Heatmap of metabolites, which were considered different between cells of *D*. *radiodurans* exposed to UVC/vacuum and non-exposed control cells. Eucledian distance was used for calculating the dendrogram. *Identification was based on database research and not on a reference substance.

Metabolite analysis from the same cells revealed 31 metabolites which were chosen for quantification. Analysis with GC-TOF usually leads to identification of primary metabolites associated with the primary metabolism. For statistical analysis, only metabolites which were present in at least three out of four replicates in at least one of the conditions were used. The normalized areas were z-scored and compared with a Welch’s t-test (p-value < 0.05). 24 metabolites, which abundances were considered different between the two conditions, were blotted as a heatmap (Fig 6). Six of them (O-Palmitoyl-L-Carnitine chloride, octadecanoic acid, ethanolamine, folic acid, mannosamine and cytidine-5-triphosphate disodium salt) were identified on level 2, all the others were identified on level 1 [25]. The majority of metabolites were more present in the control cells of *D*. *radiodurans* (S3 Table).

### Proteometabolic analysis of the TCA cycle

After exposure to stress conditions, additional energy is required to recover the cells. The TCA cycle provides large amounts of energy. Most TCA cycle related proteins showed a higher abundance in the UVC/vacuum exposed cells of *D*. *radiodurans* according to the LC-MS measurements. Accordingly, organic acids such as succinic acid, fumaric acid and malic acid were identified (at level 1) and quantified by GC-time of flight (TOF)-MS. Other metabolites were either not identified (limit of detection) or not abundant enough for quantification (limit of quantification).

**Fig 7:**
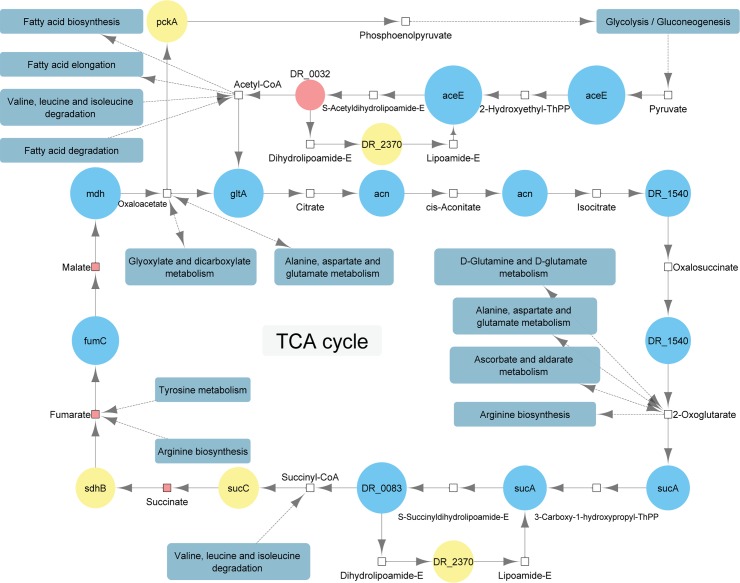
Main components of the TCA cycle in *Deinococcus radiodurans* connected to related pathways under the conditions of UVC/vacuum exposure. Metabolites are shown as rectangles. The areas of the proteins, which are shown as circles, correspond to the fold change between cells of *D*. *radiodurans* exposed to UVC/vacuum conditions and control non-exposed cells. The color shows whether the average protein or metabolite level was more abundant in the UVC/vacuum exposed cells (blue), the control cells (red), none of both conditions (yellow) or not measured/not abundant enough (colorless).

Fig 7 shows a basic version of the TCA cycle of *D*. *radiodurans* according to the KEGG website including quantitative proteomics and metabolomics data. The pyruvate dehydrogenase complex, which is responsible for the connection between glycolysis and TCA cycle as it converts pyruvate to coenzyme A, consists of three subunits. The E1 component (aceE) shows a high abundance in the irradiated cells, whereas the dihydrolipoamide acetyltransferase (DR_0032) is more abundant in the control cells. However, according to the KEGG database, another acetyltransferase DR_0256 (S2 Table), which is more abundant in the irradiated cells, is also active in the pyruvate dehydrogenase complex. The third subunit, dihydrolipoamide dehydrogenase (DR_2370) is not significantly higher abundant in any of the two conditions. Further identified and quantified proteins, which are all part of the TCA cycle, are citrate synthase (gltA), aconitate hydratase (acn), isocitrate dehydrogenase (DR_1540), 2-oxoglutarate dehydrogenase (sucA), dihydrolipoamide succinyltransferase (DR_0083), succinate-CoA ligase (sucC), succinyl-CoA synthetase (sdhB), fumarate hydratase (fumC) and malate dehydrogenase (mdh).

## Discussion

### Overall alterations in the proteome of *D*. *radiodurans* after UVC/vacuum exposure

Proteomic analysis revealed that functional categories of cysteine, methionine and tryptophan metabolism, RNA degradation, aminoacyl-tRNA biosynthesis were overrepresented in *D*. *radiodurans* exposed to UVC/vacuum conditions compared with the control cells (Fig 4). A previous study [26] showed similar categories of differentially expressed genes after gamma-irradiation. In opposite, the proteins of alanine, aspartate and glutamine metabolism were downregulated in irradiated cells of *D*. *radiodurans*. Methionine and cysteine as sulfur-containing amino acids greatly contribute to the antioxidant defense system and are key constituents in the regulation of cell metabolism. Apart from structural and catalytic role in proteins, cysteine chemistry is important to the enzymatic mechanism of the thiol-disulfide oxidoreductases of the thioredoxin superfamily, such as thioredoxins, glutaredoxin, and protein disulfide isomerase [27]. The levels of the proteins involved in cysteine biosynthesis, including thioredoxin reductase and thiosulfate sulfurtransferase, were highly upregulated in irradiated cells of *D*. *radiodurans* (S2 Table). Surface exposed methionines serve as potent endogenous antioxidants to protect other functionally essential residues from oxidative damage [28]. Methionines are readily oxidized to methionine sulfoxide by many ROS. Methionine sulfoxides can be then subsequently converted back to methionines with the help of methionine sulfoxide reductases Msr [28]. Significant upregulation (p-value 0.043) of MsrA was observed after the exposure of cells to UVC/vacuum conditions as one of the most obvious responses of *D*. *radiodurans* to oxidative damage (S2 Table, Fig 5).

The Fisher Exact test revealed a high enrichment factor for proteins connected to the TCA cycle and different amino acid pathways (Fig 4). Apart from that, RNA degradation enzymes, which dispose the damaged RNA, are enriched in the UVC irradiated under vacuum conditions cells of *D*. *radiodurans*. Finally, proteins related to the aminoacyl-tRNA biosynthesis are enriched in the irradiated cells too, indicating an increased demand of protein synthesis. These proteins catalyze the esterification of a specific amino acid to its appropriate tRNA to form an aminoacyl tRNA. In the ribosome, the amino acid is transferred from the corresponding tRNA to a growing peptide strain.

### Energy metabolism

The Fisher Exact Test indicated proteins, which are more abundant in the cells of *D*. *radiodurans* exposed to UVC/vacuum, belonging to the TCA-cycle. Nearly all key enzymes of the TCA cycle are more abundant in the cells of *D*. *radiodurans* exposed to UVC/vacuum conditions. Citrate synthase, which is responsible for the condensation of acetyl-CoA and oxaloacetate to form citrate and CoA-SH as well as aconitate hydratase, which isomerize citrate into isocitrate and isocitrate dehydrogenase, which is allosterically activated by high ADP concentrations and 2-oxoglutarate dehydrogenase (both subunits) are significantly more abundant in the irradiated cells too. Apart from succinate-CoA ligase (hydrolyse of succinyl-CoA into succinate and CoA), all other key enzymes (succinate dehydrogenase for the oxidation of succinate into fumarate, fumarate hydratase, which catalyzes the trans addition of water to produce malate and malate dehydrogenase which oxidizes malate to oxaloacetate) were found in a significantly (p-value below 0.05) higher abundance in the cells of *D*. *radiodurans* exposed to UVC/vacuum conditions (Fig 7).

Apart from enzymes directly involved into the TCA cycle, all four proteins involved in the pathway that generates pyruvate from D-glyceraldehyde 3-phosphate were more abundant in the cells exposed to UVC/vacuum. Pyruvate dehydrogenase E1 which contributes to the transformation of pyruvate to acetyl CoA for the first step in the TCA cycle was as well upregulated in the UVC/vacuum exposed *D*. *radiodurans*. Glyceraldehyde 3-phosphate can be obtained from sugars or as a by-product in the tryptophan metabolism [29]. Several proteins of the tryptophan metabolism were significantly upregulated in the UVC/vacuum exposed cells.

Daly [30] reported a higher manganese to iron ratio in *D*. *radiodurans* compared to other bacteria. Manganese contributes to the resistance against various extreme environmental conditions through the formation of ROS scavenging complexes with orthophosphate and peptides [31]. After application of these complexes, a mouse model showed increased survivability after exposure to ionizing radiation [32]. Furthermore, manganese was proposed to influence glucose incorporation into the DNA after UV exposure [33]. It was shown that glucose is solely metabolized by the pentose phosphate pathway, which augments the DNA excision repair system as it provides adequate metabolites for DNA mismatch repair [34]. Therefore, mutants which lack important pentose phosphate pathway genes, as glucose-6-phosphate-dehydrogenase (*zwf*) are more sensitive to conditions that induce DNA excision repair, such as UV irradiation [34]. Our data supports the assumption that *zwf* might participate in stress response, as it was significantly more abundant in the UVC/vacuum treated cells, which experienced DNA damage.

As there are many upregulated proteins which are directly or indirectly connected to the energy metabolism it can be assumed that more energy for regeneration is required for the UVC/vacuum exposed cells of *D*. *radiodurans* compared to the control cells. Joshi, Schmid [35] observed a degradation and resynthesis of several proteins after ionizing irradiation. The high abundance of various aminoacyl ligases in the irradiated cells indicates that the resynthesis most likely occur after the exposure to UVC/vacuum as well. The attachment of an amino acid to its tRNA which is catalyzed by these enzymes, is an energy demanding reaction which consumes one ATP per amino acid. At the same time the free amino acid pool in the UVC/vacuum exposed cells is lower than in the control cells also suggesting that protein resynthesis is a highly abundant process during recovery of the cells (Fig 6). The increased energy demand of the irradiated cells is also necessary to cope with the nucleic acid damage which is triggered by upregulation of a number of ribonucleases (S2 Table).

### DNA damage response

*D*. *radiodurans* wild type strain is approximately 25 times more resistant to UVC irradiation than *E*. *coli* wild type [11]. Early experiments with the mutagen N-methyl-N’-nitro-N-nitrosoguanidine revealed mutant strains of *D*. *radiodurans* which are more sensitive to UVC irradiation [36–38]. In 1994, Gutman, Carroll [39] confirmed a lower resistance to UVC in the *recA* and the *polA* [15] mutants. The IrrE mutant showed that the IrrE (also named *pprI*) gene function as a regulator for the expression of DNA repair and oxidative stress response proteins, like *recA* and *pprA* [40, 41]. *pprA*, which encodes a protein that can protect DNA ends from degradation and stimulate DNA-ligase activities, despite its function, seems to play a lesser role in UVC resistance, although it was upregulated in a previous UVC study [42]. Bauermeister, Bentchikou [43] showed in a comparison study that the UVC energy needed to kill 90% of a *D*. *radiodurans* culture was 1.5 times lower for the *pprA* mutant, 8 times lower for the *irrE* mutant and 20 times lower for the *recA* mutant compared to the wild type. In our proteomics analysis, *polA* (p-value 0.006) and *pprA* (p-value 0.027) were significantly more abundant in the irradiated cells, while *recA* and *irrE* levels showed no significant difference. Previous shotgun proteomic measurements of *Deinococci spp*. were primarily performed with a combination of two dimensional gel electrophoresis and MALDI-TOF after γ-irradiation. In a study conducted by Dedieu, Sahinovic [44] SSB, PprA, RecA, GyrA/B, UvrD, DdrB and DdrD showed upregulation after ionizing irradiation was applied on *Deinococcus deserti*. Another study [45] found only SSB and PprA among these proteins to be upregulated in *D*. *radiodurans* after γ-irradiation. However, in a transcriptional approach, Tanaka, Earl [22] showed an upregulation of *recA*, *gyrA/B* and also for the DNA damage response genes *ddrB* and *ddrD* in *D*. *radiodurans*. In our experiment, a higher abundance of PprA, GyrA/B (both p-values 0.002), DdrB (p-value 0.022) and DdrD (p-value 1.7*10^−4^) was observed in irradiated cells, while RecA was present constitutively at high levels in both control and irradiated cells (Fig 5, S2 Table). As our cells were incubated in TGB medium for 5 h after exposure, this fits to a kinetic study [46], which showed that RecA was upregulated for two hours after irradiation, but changed back to basal expression after four hours. Different proteomics/transcriptomics experiments showed some consistency in which DNA damage response proteins were upregulated [22, 44–46]. Differences can be explained due to a number of variable experimental parameters, e.g., dose and type of irradiation, cells dried or in suspension and recovery time. However, as shown in Fig 5, a lot of manually curated DNA damage response proteins were upregulated in our experiment, indicating that the severe DNA damage, which can be caused by UVC irradiation and desiccation stress, increases the synthesis rate of such proteins.

Contrary to the well-characterized radiation-induced damage, the strategies by which cells of *D*. *radiodurans* protect their DNA integrity in response to vacuum damage are poorly understood. Along with rapid dehydration of bacterial cells and changes in membrane permeability, DNA damage and mutagenesis have been previously described in microorganisms exposed to space vacuum [47]. Interestingly, the *gyrA* gene, coding for DNA gyrase subunit A has been reported to carry the majority of mutations induced by exposure of spores of *B*. *subtilis* to high and low vacuum [48, 49]. As suggested by our comparative proteomics analysis, GyrA protein (DR_1913) was upregulated (p-value 0.002) in UVC-irradiated cells of *D*. *radiodurans* under vacuum conditions, which can be also potentially attributed to the influence of vacuum.

Furthermore, the exposure of *D*. *radiodurans* to UVC/vacuum stress conditions triggered a suit of proteins involved in detoxification process and aimed to remove damaged nucleotides from the cell. The proteomics experiments revealed that UvrB, helixase subunit of the DNA excision repair endonuclease complex, was significantly more abundantly represented in cells of *D*. *radiodurans* in conditions of UVC/vacuum stress. The upregulated UvrB binds to DNA, searches it for potential lesions and interacts with other proteins to repair them [50]. MutT/nudix family protein (DR_0550) and MutS2 (DR_1976) involved in mismatch excision repair were upregulated in response to UVC/vacuum exposure. Some members of the Nudix family, such as MutT of *E*. *coli*, limit mutations by hydrolyzing oxidized nucleotide metabolism products, which are mutagenic once misincorporated into the genome [51, 52]. MutS2 in *D*. *radiodurans* is involved in ROS detoxification and the repair of ROS-induced DNA damage [53]. Thus, induction of Mut and Nudix family members may be one of the important protective responses to UVC/vacuum stress. Expression of the proteins (recQ and ruvABC) involved in recombinational DNA repair was also significantly induced (S2 Table).

The Mrr restriction system protein (DR_0508) that belongs to a yet unknown pathway showed high abundance (p-value 0.043) in UVC/vacuum exposed cells (S2 Table). Type IV restriction Mrr (methylated adenine recognition and restriction) endonucleases with specificity for methylated DNA have been reported to restrict DNA containing N6-methyladenine and also DNA with C5-methyl-cytosine residues [54]. Contrary to well-characterized Mcr restriction endonucleases, the physiological role of Mrr like nucleases in the cell has been less clarified. Recently, the Mrr restriction system was shown to implement into the peculiar piezophysiology of *E*. *coli*. Mrr endonuclease activity was linked to cellular filamentation and prophage induction in response to sub-lethal high-pressure shock in *E*. *coli* K12 [54, 55]. Hence, the observed up-regulation of Mrr restriction protein in *D*. *radiodurans* under the influence of UVC/vacuum conditions might assign a novel role for this less studied protein in response to space-related stress stimulus.

### Molecular systems of stress response

Our comparative proteomic analysis revealed a number of differentially abundant proteins in UVC/vacuum exposed cells of *D*. *radiodurans* that belong to the functional machinery of general stress response and oxidative stress response. Proteins of general stress response function to protect and repair damage to cellular structures, such as DNA, the cell envelope and proteins, and to provide microorganisms the ability to recuperate from the stress they experience. Overexpression of a number of chaperons occurred in UVC/vacuum exposed cells of *D*. *radiodurans*. Heat shock protein that belongs to HSP20 family (DR_1114) and chaperonins hslO (DR_0985) and groL (DR_0607), which are involved in various metabolic processes and responsible for protein folding, were upregulated in UVC/vacuum exposed cells (S2 Table). Chaperone proteins ClpB (Q9RVI3), DnaJ (Q9RUG2) and DnaK Q9RY23 were as well more abundantly represented in UVC/vacuum exposed cells of *D*. *radiodurans* (S2 Table). By binding to proteins, which are misfolded and damaged in response to various environmental stresses, these molecular chaperones can direct the misfolded proteins to the associated proteases for degradation. The elevated level of several proteases (Lon proteases Q9RXG4 and Q9RSZ5 and ATP-dependent Clp protease ClpA (DR_0588)) in irradiated cells indicates the involvement of quality monitoring and proteolytic regulation in response to combined UVC/vacuum stress.

Comparative proteomics analysis revealed a number of universal reactive oxygen species (ROS) scavengers, e.g., catalase, and redox active proteins (pyridoxal 5'-phosphate synthase, peroxidase, sulfoxide reductase MsrA, thioredoxin reductase) induced in cells of *D*. *radiodurans* exposed to UVC radiation under vacuum conditions, manifesting the upregulation of antioxidant defense mechanisms in response to these factors (Fig 5). The extreme resistance of *D*. *radiodurans* against radiation and oxidative damage relies on the high levels of constitutive catalase activity and superoxide dismutase (SOD) activity [3]. These enzymatic systems are devoted to the protection of cells against toxic reactive oxygen species. Out of three known catalases (DR1998, DRA0146, and DRA0259) in genome of *D*. *radiodurans*, our data show the elevated levels of two of them: catalase katA (p-value 1.1*10^−6^) and predicted protein with catalase function DR_A0146 (p-value 4.3*10^−4^) in UVC/vacuum exposed *D*. *radiodurans* (Fig 5). The sodA protein was constitutively represented in both irradiated and control cells (Fig 5). UVC irradiation under vacuum caused 2-fold elevated expression of the pyridoxine biosynthesis proteins PdxS and PdxT (Fig 5) which are singlet oxygen resistance proteins involved in the synthesis of vitamin B_6_, an efficient singlet oxygen quencher and a potential antioxidant [56]. The upregulated upon UVC/vacuum-irradiation thioredoxin reductase/alkyl hydroperoxide reductase (DR_1982) (Fig 5) is encoded by the gene *trxB/ahpF*, which is a key determinant of thiol redox sensing antioxidant enzymatic system in *D*. *radiodurans*. Thioredoxin reduces oxidized cysteine sulfur groups in proteins and is subsequently reverted from its oxidized form by thioredoxin reductase in an NADPH-dependent manner [3, 57]. A putative iron-dependent peroxidase (DRA_0145), enzyme that may implement in defense against oxidative stress by providing protection against toxic hydroperoxides [58], was also among upregulated proteins in response to UVC/vacuum stress. This unique putative peroxidase has very few orthologs among bacteria [58] and is listed among predicted systems of protection against oxidative stress [59].

Among significantly upregulated proteins in response to UVC/vacuum irradiation was also the peptide methionine sulfoxide reductase MsrA (DR_1849) (Fig 5, S2 Table) that shares similarity with *E*. *coli*’s methionine sulfoxide reductase and performs repair of oxidized proteins reducing protein-bound methionine sulfoxide back to methionine via a thioredoxin-recycling process [59]. Reduction of oxidized methionine residues in proteins is essential mechanism for cells survival under oxidative stress [58] and loss of MsrA sensitizes *E*. *coli* to hydrogen peroxide [60]. The induction of the gene *msrA* has been reported after ionizing irradiation of *D*. *radiodurans* [22]. Thiosulfate sulfurtransferase (DR_0217), a rhodanese superfamily enzyme was as well more abundantly represented in exposed cells of *D*. *radiodurans*. Proteins containing a single rhodanese-like domain are generally considered to mediate different forms of stress response [51]. The level of thiosulfate sulfurtransferase has been earlier reported as significantly increased after ionizing irradiation [46].

Oxidative stress-responsive proteins within tellurium resistance operon TerB (DR2220) and TerD (DR2221) were upregulated in cells of *D*. *radiodurans* exposed to UVC/vacuum conditions (Fig 5, S2 Table). The homologous tellurium resistance proteins contribute to the resistance of *E*. *coli* to various damaging agents, such as heavy metal ions and UVC radiation, and to the maintenance of the intracellular reducing environment, possibly by directly reversing disulfide bonds [3]. Several reports suggest oxidative stress as major determinant of tellurite toxicity in tellurite sensitive organisms, including *D*. *radiodurans* [61]. The genes encoding tellurium resistance have been specifically upregulated in a-proteobacterium *Rhodospirillum rubrum* followed by space exposure at ISS in frames of MELiSSA project, as well as significant differentially expressed under the conditions of modeled microgravity [62, 63]. The genes encoding TerB and TerE tellurium resistance proteins in *D*. *radiodurans* were shown to respond to acute ionizing radiation [64]. Moreover, the genes encoding TerB and TerZ proteins were found to be upregulated immediately after gamma-irradiation of *D*. *radiodurans* [22], while tellurium resistance proteins TerB and TerD were also alleviated during gamma radiation in another study [46], implementing an adaptation to oxidative stress. Apart from tellurium resistance proteins, putative copper resistance protein (DR_A0299) with the predicted function of response to stress stimulus was found more abundant in UVC-irradiated cells of *D*. *radiodurans* (S2 Table). Such an observed involvement of tellurium resistance elements in the response to radiation or factors related to space environment may be part of a metal sensing stress response system, as well as inner membrane oxidative stress response.

The red-pigmented *D*. *radiodurans* encodes a set of genes involved in biosynthesis of carotenoids [3, 51]. Carotenoid pigments have also been shown to contribute in protection against oxidative stress damage. Our comparative proteomic data analysis shows that phytoene desaturase (DR_0861), enzyme of carotenoid biosynthetic pathway in *D*. *radiodurans*, was more abundantly represented (p-value 0.043) in UVC-vacuum stressed cells (Fig 5). The arrest of lycopene synthesis and the accumulation of phytoene along with enhanced sensitivity to acute ionizing radiation and oxygen stress have been reported to the colorless DR0861 gene knockout strain, while complementation of the mutant with a heterologous or homologous gene restored pigmentation and resistance [65]. Increased abundance of phytoene desaturase in UVC-irradiated cells of *D*. *radiodurans* indicates the contribution of the carotenoid synthesis pathway to the radioresistance and oxidative stress tolerance of *D*. *radiodurans*.

Other upregulated enzymes with a possible role in oxidative stress response were probable manganese-dependent inorganic pyrophosphatase ppaC (DR_2576) involved in oxidative phosphorylation and FrnE dithiol-disulfide isomerase (DR_0659) that catalyzes formation of protein disulfide bonds and is involved in sulfur metabolism. FrnE was induced in response to ionizing radiation [22]. This thioredoxin fold protein is included in predicted radiation and desiccation resistance regulon of *Deinococci* [66].

### Transcriptional regulators

A number of transcriptional regulators and repressors have been identified in our proteomic analysis as constitutively expressed in both UVC-irradiated under vacuum conditions and control cells of *D*. *radiodurans*. The expression level of transcriptional regulators and repressors of TetR, MerR, GntR and AsnC families remained unaltered in irradiated cells of *D*. *radiodurans*, being constitutively represented under the control and UVC/vacuum conditions (S2 Table). The transcriptional regulator of FNR/CRP family (DR_0997) was significantly more abundantly represented in cells of *D*. *radiodurans* after UVC radiation under vacuum conditions. Cyclic AMP (cAMP) repressor proteins (CRP) act as global transcriptional regulators involved in many cellular pathways in various bacteria, including adaptation to starvation and extreme conditions [67–70]. The genome of *D*. *radiodurans* encodes four predicted CRP family proteins, including DR_0997, DR_1646, DR_2362, and DR_0834 (64). Recently, the gene encoding DR_0997 was shown to regulate stress response of *D*. *radiodurans* on the transcriptional level and loss of the Dr_0997 gene sensitized *D*. *radiodurans* toward H_2_O_2_, ultraviolet radiation, ionizing radiation, and mitomycin C [70]. Interestingly, our comparative proteomic analysis showed the upregulation of DR_0997 along with the upregulation of several proteins, encoded by genes, which belong to CRP regulon [70] in *D*. *radiodurans* under UVC-vacuum combined stress conditions (S2 Table). Among them are Lon proteases (DR_0349 and DR_1974), DNA repair protein PprA (DR_A0346), UvrABC system protein B UvrB (DR_2275), catalase katA (DR_0146) and tellurium resistance protein TerB (DR_2220). Thus, DR_0997 might act as a positive regulator in response to combined UVC-vacuum stress in *D*. *radiodurans*.

DdrO, a transcriptional regulator of HTH_3 family (DR_2574) was 2.6-fold downregulated in UVC-irradiated cells of *D*. *radiodurans* under vacuum conditions (S2 Table). Acting as a transcriptional repressor of Radiation Desiccation Response (RDR), DdrO binds 17 bp palindromic sequence called Radiation Desiccation Response Motif (RDRM) in 21 RDRM-promoters of *D*. *radiodurans in vitro* [71] and represses a variety of DNA Damage Response (DDR) genes. We have also found that a number of RDR proteins comprising DdrO regulon were upregulated in UVC-irradiated cells of *D*. *radiodurans* under vacuum conditions. Among them are DNA gyrase B subunit GyrB (DR_0906), Tkt transketolase (DR_2256), RecQ helicase (DR_1289), UvrD superfamily I helicase (DR1775), urocanate hydratase (DRA0151) and FrnE uncharacterized DsbA-like thioredoxin fold protein (DR_0659) (S2 Table). Apparently, DdrO as a global master regulator serves to control reprogramming of microbial physiology in order to permit the adaptation of *D*. *radiodurans* to combined UVC-vacuum stress.

### Metabolic regulation

Our approach focuses on the identification and quantification of polar, primary metabolites. These are involved in growth, development and reproduction—parameters which are affected by UVC/vacuum stress. The metabolite analysis showed a significantly reduced abundance of overwhelming majority of identified polar metabolites in the irradiated cells of *D*. *radiodurans* (Fig 6). As *D*. *radiodurans* is a bacterium with a proteolytic life-style, it uses amino acids as preferred carbon source [72, 73]. Ethanolamine was one of the very few metabolites, which were more abundant in the UVC-irradiated cells of *D*. *radiodurans* (Fig 6). Splitting ethanolamine into ammonia and acetaldehyde can serve as a cellular supply of reduced nitrogen as well as a precursor for acetyl CoA [74], sustaining the necessary levels of these compounds in irradiated cells. Interestingly, the elevated level of a palmitoyl-derivative of carnitine was observed in the UVC-vacuum exposed cells of *D*. *radiodurans* (Fig 6). Apart from its nutritional function, a quaternary amine compound carnitine has various physiological effects. As a compatible solute, carnitine is important osmoprotectant, and can also enhance thermotolerance, cryotolerance and barotolerance, impacting bacterial survival in extreme conditions [75]. At the same time, osmotic stress has been described as a part of stress response which microorganisms experience exposed to the outer space environment or to its individual simulated factors [62, 76–78] In this context, the observed upregulation of O-Palmytoyl-L-Carnitine chloride (Fig 6) may suggest the role of this quaternary amine compound responsible for adaptation to extreme conditions [70] in the protection of *D*. *radiodurans* against combined stress conditions of UVC and vacuum. Moreover, carnitine as a compatible solute might be potentially necessary to overcome damaging desiccation effects of vacuum [47, 79] by binding additional water molecules, helping to stabilize proteins and cell membranes, and thus preventing complete desiccation of the cell.

The amount of octadecanoic (stearic) acid, which has been described as a minor component of *D*. *radiodurans* [75], was significantly increased in vacuum/UVC-irradiated cells of *D*. *radiodurans* (Fig 6). The surface-active compound stearic acid was identified in biosurfactants of several bacterial species [76, 77]. Decreased levels of stearic acid associated with the dramatic reduction in biofilm formation of *Streptococcus sanguinis nox* mutant [78], suggesting its involvement in stress-related reactions. Stearic acid can potentially be involved in covering the cells of *D*. *radiodurans* by a layer less permeable to water, thereby preserving the structural integrity of cell membranes in conditions of vacuum-induced dehydration. Although the cells of *D*. *radiodurans* do not naturally produce stearic acid in big quantities under non-stressed conditions [75], the observed accumulation of this biofilm-associated compound may potentially lead to the high survival of *D*. *radiodurans* in dry multilayers under UVC/vacuum combined stress.

**Fig 8:**
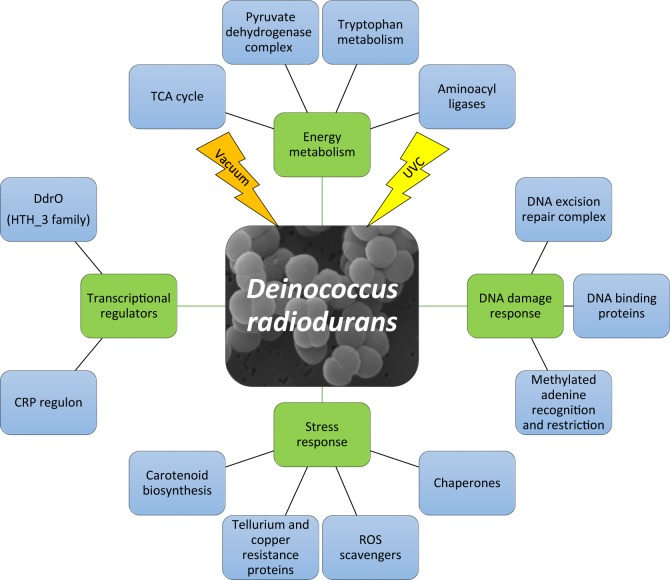
Molecular response of *D*. *radiodurans* experienced under UVC and vacuum conditions. First two levels of molecular pathways are represented which are affected by UVC irradiation under vacuum conditions.

Our study shows that response to UVC/vacuum combined stress and the enzymatic repair caused by the damage after ionizing radiation have overlapping molecular components in *D*. *radiodurans*. The combination of proteomic with metabolomic analysis of cells after UVC-irradiation under vacuum condition reveals that the response is a multilayer process (Fig 8). It requires a high amount of energy in order to initiate stress defense mechanisms necessary to alleviate cell damage.

## Supporting information

S1 Fig Scanning electron micrographs showing multilayers of dehydrated cells of *D*. *radiodurans* deposited on aluminum plates and used in experimental set up of Tanpopo mission. A, control cells of *D*. *radiodurans* dried in aluminum plates in accordance to Kawaguchi et al., 2016. B, dried cells of *D*. *radiodurans* after exposure to UVC/vacuum conditions. Shown is the upper surface of dehydrated *D*. *radiodurans* multilayers.
(TIF)

S1 Table Individual and average relative survival rates for four control and irradiated replicates. (XLSX)

S2 Table Raw proteomic data. (XLSX)

S3 Table Raw metabolomic data. (XLSX)
