# Supplementary figures and images for: Proteometabolomic response of Deinococcus radiodurans exposed to UVC and vacuum conditions: Initial studies prior to the Tanpopo space mission
Source: PLoS One. 2017 Dec 15;12(12):e0189381. doi: 10.1371/journal.pone.0189381 (PMC5731708; doi:10.1371/journal.pone.0189381)

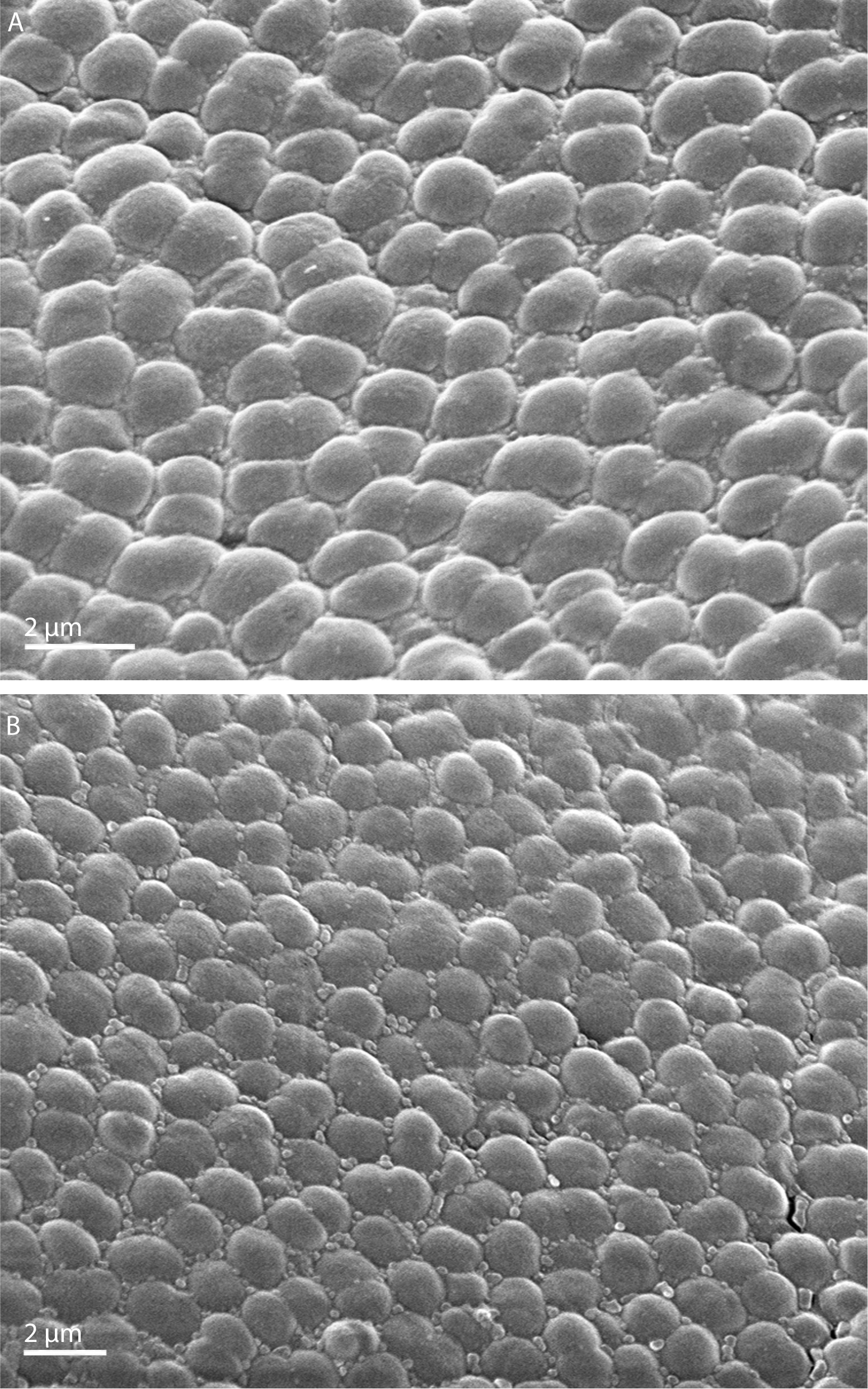

Supplement: S1 Fig — A, control cells of D. radiodurans dried in aluminum plates in accordance to Kawaguchi et al., 2016. B, dried cells of D. radiodurans after exposure to UVC/vacuum conditions. Shown is the upper surface of dehydrated D. radiodurans multilayers. (TIF) [file pone.0189381.s001.tif]
